# Heart rate variability as an indicator of left ventricular systolic dysfunction

**Published:** 2009-10

**Authors:** Abdulla Shehab, Asim A Elnour, Allan D Struthers

**Affiliations:** Department of Internal Medicine, Faculty of Medicine and Health Sciences, United Arab Emirates University; Medical Institute, Al Ain Hospital in affiliation with VAM ED and Medical University of Vienna, Abu Dhabi Health Services Company; Pharmacy Department and Medical Institute, Al Ain Hospital in affiliation with VAM ED and Medical University of Vienna, Abu Dhabi Health Services Company, Al Ain, United Arab Emirates; Cardiovascular Medicine and Therapeutics, Department of Clinical Pharmacology and Therapeutics, Ninewells Hospital and Medical School, Dundee, Scotland

## Abstract

**Objectives:**

The aim was to compare measures of heart rate variability (HRV) in patients who presented with non-cardiac vascular episodes with age- and gender-matched control patients.

**Methods:**

One hundred and fifty patients, randomly selected from a cohort of 522 subjects, were enrolled in a screening study. Of these, 256 were identified to have had a stroke or transient ischaemic attack (TIA), or to have peripheral vascular disease (PVD) at the first presentation to Ninewells Hospital, Dundee, Scotland. Only 114 patients remained in the study (100 cases and 14 controls). Multiple regression analysis was used to assess the association between HRV parameters and measures of mean heart rate and ejection fraction.

**Results:**

Heart rate and HRV indices were significantly inversely correlated with both normal left ventricular (LV) function [*r* = 0.2–0.5; *p* = 0.037–0.0001] and left ventricular systolic dysfunction (LVSD) [*r* = 0.3–0.5; *p* = 0.07–0.01] in the patients. HRV did not predict LVSD in this cohort of patients. Multiple regression analysis showed only ischaemic heart disease (IHD) and cigarette smoking had an independent relation to HRV parameters. Cigarette smoking (*p* = 0.008), IHD (*p* = 0.02) and diabetes (*p* = 0.03) were significant predictors of reduced HRV (standard deviation of the normal-to-normal interval: SDNN), independent of LVSD.

**Discussion:**

There were no significant differences in HRV indices between non-cardiac vascular patients (TIA, stroke, PVD) and their age- and gender-matched controls. HRV had no diagnostic value as a pre-screening test to identify suspected LVSD in these patients.

**Conclusion:**

HRV cannot be used as a screening test to identify hidden LVSD. Further studies will be needed to assess the possibilities that HRV is a convenient marker of endothelial dysfunction.

## Summary

Heart rate variability (HRV) is a non-invasive index of the autonomic function of the heart. Abnormal cardiac autonomic function may be an important contributor to the pathophysiology of vascular disease, heart failure and myocardial ischaemia and their consequences, in particular sudden cardiac death.

In patients with left ventricular systolic dysfunction (LVSD), a reduced standard deviation of the normal-to-normal interval (SDNN) of heart rate variability was found to be an independent predictor of cardiac death but not sudden death in outpatients in the UK-Heart trial in 1998.^1^ In patients with more severe heart failure, reduced HRV was independent of left ventricular ejection fraction (LVEF) and the occurrence of ventricular tachycardia (VT).^2^,^3^ In patients with myocardial infarction (MI) and LVSD, depressed SDNN and LVEF both independently predicted cardiac death.^4^,^5^

In stroke patients, distorted HRV predicted a poor outcome.^6^ The relationship between stroke and depressed HRV is intriguing when considering that patients are at a high risk of sudden death in the first month,^7^ with increasing possibility of dying from cardiac death within the first year.

In stroke patients, hemispheric brain infarction causes longstanding damage to the cardiovascular autonomic regulatory system.^8^,^9^ Sympathetic tone is increased and parasympathetic function is impaired, both of which directly affect cardiac autonomic function.^10^,^11^ Since an imbalance in cardiac autonomic innervation may be crucial for the generation of cardiac arrhythmias and reduced HRV has been associated with increased mortality, Naver and co-workers suggested that the risk of sudden death may be correlated with lateralisation and location of the brain infarct after stroke (left or right hemisphere stroke).^12^

Reduced HRV might be an important tool to risk-stratify patients who are at risk of developing sudden cardiac death. It is already established that drugs such as beta-blockers, angiotensin converting enzyme (ACE) inhibitors and amiodarone have a favourable influence on HRV and this corresponds with a reduction in cardiac mortality.^13^

The aim of this study was to delve deeper into the subject of HRV in patients who presented with non-cardiac vascular episodes, and in particular to compare the measures of HRV in such patients, with age- and gender-matched control patients who had been recruited for the initial screening study. We also wanted to evaluate whether reduced HRV could be used as a screening test to help identify patients with LVSD, in order to develop a non-invasive measure (HRV) to assist in predicting sudden death or LVSD in subjects. In addition, we wanted to identify which underlying cardiovascular abnormalities (e.g. LV dysfunction) were associated with a reduced HRV in this cohort of patients.

## Methods

One hundred and fifty patients were enrolled in the study after being randomly selected from a cohort of subjects (*n* = 522) who had enrolled in a screening study. Of these, 256 were identified to have had a stroke or transient ischaemic attack (TIA) or had peripheral vascular disease (PVD) at first presentation to Ninewells Hospital, Dundee, Scotland.

One hundred and twenty non-cardiac vascular disease patients (stroke, TIA and PVD) and 30 age- and gender-matched controls agreed to participate in the ambulatory substudy of the main study. These patients had similar demographic characteristics to those of the original screening population. Patients with atrial fibrillation or flutter, pacemaker implantation, poor-quality holter recordings and those with echocardiographic images of inadequate quality were excluded. There were 36 patients subsequently excluded because of the following: 16 had poor-quality holter recordings, 12 had suboptimal echo images, five had atrial fibrillation and three had left bundle branch block.

Only 114 patients remained in the study (100 cases and 14 controls), 62 were males and 52 females. The cases were distributed as follows: 24 stroke, 40 TIA and 36 PVD patients. Stroke patients were identified from a hospital stroke admission database and this diagnosis was confirmed with a CAT scan.

Patients with TIA included only those with classic symptoms. These patients were recruited from referrals to the vascular laboratory for carotid ultrasound. Their clinical history determined inclusion in the study cohort and not their ultrasound findings. Patients with vague symptoms, or a referral for investigation of dizzy spells/funny turns were excluded.

PVD patients were also recruited from the vascular laboratory, based on the history of new-onset intermittent claudication in conjunction with an ankle–arm index of < 0.8 at rest. PVD patients with acute leg ischaemia were excluded.

Vascular patients with a previous stroke, TIA or PVD were excluded, as this would constitute their second non-cardiac vascular event. A history of previous MI or of coronary artery disease did not preclude any patient from being recruited.

Age- and gender-matched control subjects were recruited from the general population. These patients differed from the case group solely on the absence of previous stroke, TIA or history of PVD. The cases were matched for age and gender using our own hospital medicines monitoring unit (MEMO) database, which represents the general population. Patients with stroke, TIA or PVD were removed from the control list.

Each patient underwent a full history and physical examination. The history included a questionnaire on symptoms, cardiac risk factors, previous hospital admission, current medications, age, gender and details of presenting vascular event. Blood pressure was recorded with an automated sphygmomanometer. Each reading was taken as an average of three recordings from both arms with the patient seated, after five minutes’ rest.

Blood samples were collected for urea and electrolyte analysis and brain natriuretic peptide (BNP).

## Echocardiographic examination

M-mode and two-dimensional echocardiographic examinations were performed. A single observer who was blinded to the patient’s clinical characteristics performed all echocardiograms. Two-dimensional echocardiography (HP Sonos 2000) was performed with each patient reclining in a lateral position at 45 degrees. Images were stored on videotape and analysed.

The LVEF was measured by the biplane disc summation method (modified Simpson’s rule). LVSD was defined as LVEF < 40%. Each ejection fraction was taken as a mean of three cardiac cycles. Echocardiograms were deemed acceptable if 75% or more of the endocardium was visible.

## Electrocardiographic measurements

Standard 12-lead electrocardiograms were performed using a Marquette MAC eight-lead electrocardiogram machine (Palo Alta, California, USA). Each anonymous ECG was then analysed at a later date. The ECG was reported as normal or abnormal. An abnormal ECG was defined by the presence of atrial fibrillation, atrial flutter, left bundle branch block, left ventricular hypertrophy, pathological Q-waves and ischaemia. Ischaemia included ST-segment depression, any T-wave inversion and Q-waves. Unresolved ECGs were resolved by a second observer.

HRV measures were derived from 24-hour electrocardiography monitoring calculated in the time domain. Analysis of HRV was performed according to standard guidelines.^14^ Time-domain measures included the overall variability in the entire recording, calculated as the standard deviation (SD) of all normal R–R intervals (SDNN).

Another time-domain measure that reflects the overall HRV in the entire recording is the triangular index. This is a geometric measure obtained by dividing the total number of all R–R intervals by the height of the histogram of all R–R intervals, measured on a discrete scale with bins of 7.8 ms. The height of the histogram equals the total number of intervals found in the modal bin.

Long-term HRV was estimated using the standard deviation of the mean R–R values from all five-minute segments (SDANN) and the mean of the standard deviations of all normal sinus R–R intervals for all five-minute segments (SDNN index). Short-term HRV in five-minute periods was measured using the SDNN index, a mean of five-minute standard deviations of R–R intervals.

ECG data was summarised as a mean for an individual patient across 24 months of study visits. Hourly values were corrected to a 24-hour mean value where relevant. Cut-off values of < 100 ms for SDNN and < 25 ms for triangular index (TI) were used where appropriate.

## Statistical analysis

An SPSS version 10 was used for statistical analysis. All results were expressed as means and standard error of the means (SEM). A two-sample t-test was used to compare differences between the two groups. To determine the relationship between HRV and other measured variables, univariate analysis was done, followed by multiple regression analysis in a step-wise fashion if variables demonstrated a significant relationship with HRV. Multiple regression analysis was used to assess the association between HRV parameters and measures of mean heart rate and ejection fraction. A *p*-value < 0.05 was considered to be significant.

## Results

The baseline characteristics of the cases and controls are illustrated in Table 1. Both groups were matched in terms of serum electrolytes, cardiac medication, LV function and cardiac risk factors. Thirty-eight per cent of cases and 14% of controls had symptoms of LVSD. The mean age was 69 years for both groups.

**Table 1:** Baseline Characteristics Of Cases And Control Patients

*Risk factor*	*Cases (%)*	*Control (%)*	p*-value*
Mean age (years)	69	69	NS
Female gender	47 (47)	5 (22)	0.03
Symptoms	38 (38)	2 (14)	0.04
Mean SBP (mmHg)	160 (2.6)	159 (8.2)	NS
Mean DBP (mmHg)	88 (3.2)	84 (1.2)	NS
Mean HR (beats/min)	79 (1.5)	79 (3.5)	NS
MI/IHD	33 (33)	2 (14)	0.09
DM	14 (14)	0	-
BP	41 (41)	5 (38)	NS
Hyperlipidaemia	43 (43)	1 (7)	0.0003
Smoking	80 (80)	11 (79)	NS
Aspirin	65 (65)	2 (14)	0.0001
Beta-blockers	17 (17)	2 (14)	NS
ACE inhibitors	16 (16)	2 (14)	NS
Statins	27 (27)	1 (7)	0.025
Mean K (mmol/l)	4.16 (0.04)	4.06 (0.16)	NS
Mean Na (mmol/l)	139.3 (0.3)	136.7 (2.1)	NS
Mean BNP (pmol/l)	64.1 (6.5)	42.4 (11)	NS
Mean EF %	44 (0.9)	47 (2.7)	NS

NS: non significant (*p* > 0.05), SBP: systolic blood pressure, DBP: diastolic blood pressure, HR: heart rate, MI: myocardial infarction, IHD: ischaemic heart disease, DM: diabetes mellitus, BP: blood pressure, ACE: angiotensin converting enzyme inhibitor, K: potassium, Na: sodium, BNP: brain natriuretic peptide, EF: ejection fraction.

Table 2 shows that 30 patients had LVSD and 70 had normal LV function. The groups were matched for age, presence of TIA, stroke, PVD, potassium levels and cardiac risk factors (except for IHD). LVSD patients were more likely to be men, with a previous history of MI/IHD, who were taking diuretics, ACE inhibitors, beta-blockers and aspirin (*p* < 0.05). Fifty-seven per cent of patients with LVSD were asymptomatic. There was no significant difference in systolic and diastolic pressure between the group of patients with and without LVSD (*p* > 0.05). There was no significant correlation between ejection fraction and HRV indices.

**Table 2:** Baseline Characteristics For Patients With And Without LVSD

*Risk factor*	*Normal LV (n = 70)*	*LVSD (n = 30)*	p*-value*
Mean age (years)	69	69	NS
Male gender	42 (60)	20 (66.7)	0.04
Symptoms	25 (36)	13 (43)	NS
TIA	30 (43)	10 (33)	NS
Stroke	17 (24)	7 (23)	NS
PVD	23 (33)	13 (043)	NS
MI/IHD	17 (24)	16 (53)	0.008
DM	7 (10)	7 (23)	NS
Hypertension	29 (41)	12 (40)	NS
Smoker	54 (77)	26 (86)	NS
Hyperlipidaemia	30 (43)	13 (43)	NS
ACE inhibitors	6 (9)	10 (33)	0.01
Diuretics	10 (14)	11 (36)	0.024
Beta-blockers	15 (21)	2 (7)	0.038
Statins	20 (29)	7 (23)	NS
Aspirin	41 (59)	24 (80)	0.011
BNP (pmol/l)	51 (5.5)	95.7 (16)	0.01
Mean SBP (mmHg)	158 (2.8)	167 (5.5)	NS
Mean HR beats/min)	78 (1.7)	72 (2.6)	NS

LV: left ventricle, LVSD: left ventricular systolic dysfunction, NS: not significant (*p* > 0.05), TIA: transient ischaemic attack, PVD: peripheral vascular disease, MI: myocardial infarction, IHD: ischaemic heart disease, DM: diabetes mellitus, ACE: angiotensin converting enzyme inhibitor, BNP: brain natriuretic peptide, SBP: systolic blood pressure, HR: heart rate.

Heart rate and HRV indices were significantly inversely correlated with both normal LV function (*r* = 0.2–0.5; *p* = 0.037–0.0001) and LVSD (*r* = 0.3–0.5; *p* = 0.07–0.01). HRV parameters of the patients were reasonably different from those of the controls but these changes did not reach statistical significance (*p* > 0.05). The results show that the patients with LVSD had reduced (but not statistically significant) resting heart rates and HRV parameters (SDNN and SDANN). HRV did not predict LVSD in this cohort of patients. The results are given in Tables 3 and 4.

**Table 3:** HRV Analysis In Case And Control Patients

*HRV (ms)*	*Case mean (SEM)*	*Control mean (SEM)*	p*-value mean (SEM)*
SDANN	103 (3)	120 (11)	NS
RMSSD	28 (2.0)	22 (2)	NS
Triangular index	29 (1.0)	35 (3)	NS
SDNN	112 (3)	129 (11)	NS
SDNN index	44 (2)	45 (4)	NS

SEM: Standard error of mean, NS: not significant (*p* > 0.05), SDANN: standard deviation of average normal-to-normal interval, RMSSD: mean square root of successive differences, SDNN: standard deviation normal-to normal interval.

**Table 4:** HRV In Case Patients With And Without LVSD

*HRV (ms)*	*Normal LV mean (SEM)*	*LVSD mean (SEM)*	p*-value mean (SEM)*
RMSSD	27 (2)	31 (4)	NS
Triangular index	29 (1)	28 (2)	NS
SDNN	113 (4)	109 (7)	NS
SDNN index	44 (3)	43 (2)	NS
SDANN	104 (4)	100 (6)	NS

LV: Left ventricle, LVSD: left ventricular systolic dysfunction, SEM: standard error of mean, NS: not significant (*p* > 0.05), RMSSD: mean square root of successive differences, SDNN: standard deviation normal-to-normal interval, SDANN: standard deviation average normal-to-normal interval.

Multiple regression analysis was applied to all 11 variables (age, gender, LV dysfunction, cigarette smoking, hyperlipidaemia, diabetes mellitus, hypertension, IHD/MI, serum sodium and serum potassium). The results indicate that of these variables, only IHD and cigarette smoking showed an independent relation to HRV parameters as follows: IHD was non-significantly related to SDNN (*p* = 0.06); cigarette smoking was significantly related to SDNN (*p* = 0.007) and both were marginally related to HRV triangular index (*p* = 0.085; Table 5).

**Table 5:** Multiple Regression Analysis Of The Risk Factors That Reduce HRV In Non-Cardiac Vascular Case Patients Independent Of LV Function

*HRV (ms)*	*All case patients*	*LVSD cases*	*Normal LV cases*
Triangular index	Smoking	N/A	Smoking
SDNN	*MI/IHD	N/A	Potassium
SDNN index	Smoking	N/A	N/A
SDANN	N/A	N/A	Diabetes
RMSSD	N/A	N/A	N/A

LVSD: left ventricular systolic dysfunction, LV: left ventricle, SDNN: standard deviation normal-to-normal interval, N/A: not applicable, SDANN: standard deviation average normal-to-normal interval, RMSSD: mean square root of successive differences, *p* = 0.06 for *MI/IHD, MI/IHD: myocardial infarction/ischaemic heart disease, other risk factors in analysis had *p*-values < 0.05.

In the non-cardiac vascular patients with LVSD, no cardiac risk factor was related to HRV. However, in patients with normal LV function the following variables showed a trend towards reduced HRV: serum potassium was related to SDNN (*p* = 0.03), diabetes mellitus was related to SDANN (*p* = 0.044) and cigarette smoking was marginally related to HRV triangular index (*p* = 0.07). Cigarette smoking (*p* = 0.008), IHD (*p* = 0.02) and diabetes (*p* = 0.03) were significant predictors of reduced HRV (SDNN), independent of LVSD.

Drug therapy was evaluated in the patient group using a separate multiple regression analysis. Six drugs (aspirin, ACE inhibitors, diuretics, digoxin, beta-blockers and statins) were entered into the analysis. Statin therapy significantly (*p* < 0.05) but inversely related to some indices of HRV (SDANN, triangular index and SDNN; Table 6). Of all these medications, digoxin and beta-blockers had a favourable relation, but not statistically significant, with HRV indices in the non-cardiac vascular patients (*p* > 0.05; Table 7, 8).

**Table 6:** Association Of Statin Therapy With Mean HRV In Case Patients

*HRV (ms)*	*Statin therapy*	*No statin*	p*-value*
SDNN	100.4	116.4	0.04
Triangular index	25	30	0.05
SDNN index	40	45	NS
RMSSD	24	29	NS
SDANN	91	107.4	0.031

SDNN: standard deviation normal-to-normal interval, NS: not significant (*p* > 0.05), RMSSD: mean square root of successive differences, SDANN: standard deviation average normal-to-normal interval.

**Table 7:** Association Of Digoxin Treatment With Mean HRV In Case Patients

*HRV (ms)*	*Digoxin*	*No digoxin*	p*-value*
SDNN	132	111	NS
Triangular index	34	29	NS
SDNN index	87	42	NS
SDANN	106	163	NS
RMSSD	59	27	NS

SDNN: standard deviation normal-to-normal interval, NS: not significant (*p* > 0.05), SDANN: standard deviation average normal-to-normal interval, RMSSD: mean square root of successive differences.

**Table 8:** Association Of Beta-Blockers With HRV Indices In Case Patients

*HRV( ms)*	*Beta-blockers*	*No beta-blockers*	*p-value*
SDNN	110	112	NS
Triangular index	27	29	NS
SDNN index	68	44	NS
SDANN	106	102	NS
RMSSD	27	32	NS

SDNN: standard deviation normal-to-normal interval, NS: not significant (*p* > 0.05), SDANN: standard deviation average normal-to-normal interval, RMSSD: mean square root of successive differences.

There was no significant difference in HRV between males and females (*p* > 0.05; Table 9) despite a deteriorating trend in some of the HRV indices [SDNN, TI and square root of the mean of squared differences between adjacent normal R–R intervals (RMSSD)]. The association between age and HRV in this study was opposite to what one would expect. Older patients (age > 60 years) had significantly higher HRV (*p* < 0.05) than younger patients, which applied to all HRV indices (Table 10).

**Table 9:** Relationship Between HRV And Gender In Case Patients

*HRV (ms)*	*Male*	*Female*	p*-value*
SDNN	114 (5)	110 (5)	NS
Triangular index	31 (1.5)	27 (1)	NS
SDNN index	45 (3)	42 (3)	NS
SDANN	102 (4)	103 (5)	NS
RMSSD	29 (3)	27 (2)	NS

SDNN: standard deviation normal-to-normal interval, NS: not significant (*p* > 0.05), SDANN: standard deviation average normal-to-normal interval, RMSSD: mean square root of successive differences.

**Table 10:** Relationship Between HRV And Age In Case Patients

*HRV (ms)*	*Age < 60 years*	*Age > 60 years*	p*-value*
SDNN	106 (4)	136 (8)	0.004
SDNN index	38 (1)	71 (8)	0.0009
SDANN	99 (4)	118 (9)	0.05
RMSSD	24 (2)	45 (5)	0.0002
Triangular index	28 (1)	33 (2)	0.048

SDNN: standard deviation normal-to-normal interval, SDANN: standard deviation average normal-to-normal interval, RMSSD: mean square root of successive differences.

With regression analysis, we used the recommended cut-off values for HRV prior to this study: SDNN < 100 ms and triangular index < 25 ms, which identified the case patients (*n* = 63, *n* = 23), respectively. SDNN < 100 ms was significantly related to a previous history of MI and IHD (*p* < 0.043) and statin therapy was associated with an SDNN > 100 ms (*p* = 0.06). The triangular index cut-off value < 25 ms was significantly related to the male gender (*p* = 0.032) and previous MI/IHD (*p* = 0.033). In this respect, diuretic (*p* = 0.002) and statin therapy (*p* = 0.028) favoured a triangular index > 25 ms (Table 11).

**Table 11:** HRV Cut-Off Values And Cardiac Risk Factors Regression Analysis

*HRV variables*	*Risk factors*	p*-value*
SDNN (ms) < 100	MI/IHD	0.043
SDNN (ms) >100	Statin therapy	0.06
Triangular index < 25	Male gender	0.032
	MI/IHD	0.033
Triangular index > 25	Statin therapy	0.028

MI/IHD: myocardial infarction/ischaemic heart disease, SDNN: standard deviation normal-to-normal interval, SDANN: standard deviation average normal-to-normal interval, RMSSD: mean square root of successive differences.

SDNN < 100 ms and a triangular index < 25 ms were evaluated for identifying non-cardiac vascular patients with IHD and LVSD.

● For LVSD, SDNN < 100 ms had an area under the curve (AUC) of 0.49, a sensitivity of 30% and a specificity of 70%. Triangular index < 25 ms had an AUC of 0.559, with a sensitivity of 39% and a specificity of 73%.
● For IHD/MI, SDNN < 100 ms had an AUC under the receiver operator curve (ROC) of 0.608, with a 52% sensitivity and a 71% specificity. Triangular index < 25% had an AUC of 0.62, with 39% sensitivity and 85% specificity. Other cut-off points yielded AUC under the ROC < 0.5, with poor sensitivity.

## Discussion

This study showed that there were no significant differences in HRV indices between non-cardiac vascular patients (TIA, stroke, PVD) and their age- and gender-matched controls. In fact, the lower HRV values and high resting heart rate, which were coupled with the LVSD, though not significant in this study, confirmed the neurohormonal pathophysiology of chronic heart failure. This is due to complex autonomic regulation and the involvement of the parasympathetic nervous system.^15^

The sensitivity of HRV in identifying patients with LV dysfunction was very poor despite using the recommended cutoff values of < 100 ms for SDNN and < 25 ms for TI, not making HRV a useful screening tool for LV dysfunction. From our study, HRV had no diagnostic value as a pre-screening test to identify suspected LVSD in TIA, stroke or PVD patients.

Stroke and heart failure data showed that HRV was reduced in non-cardiac vascular patients and those with LVSD.^5^,^7^ In view of the link between HRV and nitric oxide concentrations, the question arises whether HRV could be explained by endothelial dysfunction. We know that LVSD is associated with endothelial dysfunction and reduced HRV may be due to atherosclerosis, coronary artery disease, diabetes and cigarette smoking, all of which produce abnormal endothelial function.

The link between HRV and endothelial dysfunction was seen in Choudhury’s work,^16^ which showed that endogenous nitric oxide (NO) can augment vagal control of the heart rate in humans. Therefore, the main hallmark of endothelial dysfunction is reduced activity of vascular nitric oxide.^17^ NO deficiency results in autonomic and baroreflex dysfunction, which produces a reduction in the vagal component of HRV.^18^ Hence, endothelial function and autonomic function (as evidenced by HRV) are likely to be closely linked because NO is an important determinant of both.

Many pathological states, such as heart failure, hypertension, diabetes, hypercholestrolaemia, atheroma and cigarette smoking have been associated with abnormalities in the generation or function of NO. These conditions are also associated with both autonomic and endothelial dysfunction.^19^

In our study, reduced HRV was associated with gender and age, although it was lower in younger people, contrary to the findings of the Framingham study. This showed that older age reduced HRV in the general population.^20^ However, Fauchier and co-workers reported that young patients under 50 years with dilated cardiomyopathy had reduced HRV, which was an adverse prognosis in these patients.^21^ In the ATRAMI study,^5^ age did not influence HRV, but younger patients with low HRV had a poorer prognosis following MI. The disparate findings of our population compared to previous work, particularly with regard to older subjects who had a higher HRV than younger patients, could indicate a subset of the general population.

Could reduced HRV in this study be associated with endothelial dysfunction? Reduced HRV in patients with LVSD was associated with a history of IHD/MI and cigarette smoking, and in patients with normal LV function this was associated with low potassium levels, smoking and diabetes. Each of these risk factors could cause endothelial dysfunction and this might explain why they were associated with reduced HRV in these non-cardiac vascular patients. These patients might already have had endothelial dysfunction, which had caused their stroke, TIA or PVD in the first place. Hence the depressed HRV may reflect accelerated endothelial dysfunction and disease progression. Further evaluation is required to confirm or refute the hypothesis that endothelial dysfunction may explain in part why HRV was reduced in the TIA, stroke and PVD patients.

Unfortunately, this cross-sectional study was not able to discover the reason for so many sudden deaths in the noncardiac vascular patients with low HRV. On the basis of this and other studies, one can speculate from the available results that reduced HRV is independent of LVSD, but it may be an indicator of endothelial dysfunction. This may indicate why HRV was an independent predictor of cardiac death in these patients. Although HRV was not a marker for unsuspected LVSD, it may be an independent culprit promoting cardiac death, as it could be a marker for endothelial dysfunction.

In stroke patients, no direct link between reduced HRV and cardiac mortality has yet been established. This is despite the existence of evidence of such an association between reduced HRV and increased mortality in many other groups of patients.

In the analysis, we found a favourable effect of beta-blockers and statin therapy on HRV indices, which was consistent with previous studies.^22^-^26^ These drugs are mostly known to reduce mortality, which increases the association between HRV and cardiac death at least for beta-blockers and statins. From previous studies we know that digoxin does not reduce cardiac mortality despite enhancing HRV.^27^ On the other hand, statins showed a reduction in HRV and were known to improve mortality in well-controlled randomised studies.^28^,^29^ It is possible that digoxin has other unfavourable effects that counteract its favourable effects on HRV.

## Limitations

Despite using age and gender-matched control patients, some subjects did have heart disease and were on drug therapy, which might have complicated the results. In the non-cardiac vascular group with LVSD, IHD/MI and cardiac medications may possibly have influenced the findings. The regression analysis attempted to correct for these differences, but it can never fully correct for all influences. Another crucial issue was that patients in this study did not undergo coronary angiography. One cannot rule out the possibility that the prevalence of coronary artery disease may have been higher than we found from clinical history taking alone. The small sample size might have contributed in part to the non-significant results. This implies that clinically significant findings may have been obtained with a larger number of patients.

## Conclusions

Twenty-four HRV analyses using time-domain indices in noncardiac vascular patients did not correlate with LVSD. The first implication is that HRV cannot be used as a screening test to identify hidden LVSD. Secondly, the link between HRV and mortality was not because HRV and LVSD clustered together. This raised the possibility that HRV was a culprit in itself rather than a marker of hidden LVSD. However, it is equally possible that HRV and endothelial dysfunction clustered together and that HRV was a convenient signal of endothelial dysfunction. The latter is itself a poor prognostic indicator because it predicts further thrombolytic events on top of the dysfunctional (sticky) endothelium. Further studies are needed to assess the possibility that HRV is a convenient marker of endothelial dysfunction.
